# Computational Prediction of the Interaction of Ivermectin with Fibrinogen

**DOI:** 10.3390/ijms241411449

**Published:** 2023-07-14

**Authors:** Paola Vottero, Scott Tavernini, Alessandro D. Santin, David E. Scheim, Jack A. Tuszynski, Maral Aminpour

**Affiliations:** 1 Department of Biomedical Engineering, University of Alberta, Edmonton, AB T6G 1Z2, Canada; vottero@ualberta.ca (P.V.); aminpour@ualberta.ca (M.A.); 2 Department of Mechanical Engineering, University of Alberta, Edmonton, AB T6G 1H9, Canada; staverni@ualberta.ca; 3 Obstetrics, Gynecology & Reproductive Sciences, Yale School of Medicine, P.O. Box 208063, New Haven, CT 06520-8063, USA; alessandro.santin@yale.edu; 4 US Public Health Service, Commissioned Corps, Inactive Reserve, Blacksburg, VA 24060-6367, USA; dscheim@alum.mit.edu; 5 Department of Physics, University of Alberta, Edmonton, AB T6G 1Z2, Canada; 6 DIMEAS, Politecnico di Torino, 10129 Turin, Italy; 7 Department of Data Science and Engineering, The Silesian University of Technology, 44-100 Gliwice, Poland

**Keywords:** fibrinogen, microclots, docking, molecular modeling, SARS-CoV-2, ivermectin

## Abstract

Hypercoagulability and formation of extensive and difficult-to-lyse microclots are a hallmark of both acute COVID-19 and long COVID. Fibrinogen, when converted to fibrin, is responsible for clot formation, but abnormal structural and mechanical clot properties can lead to pathologic thrombosis. Recent experimental evidence suggests that the spike protein (SP) from severe acute respiratory syndrome coronavirus 2 (SARS-CoV-2) may directly bind to the blood coagulation factor fibrinogen and induce structurally abnormal blood clots with heightened proinflammatory activity. Accordingly, in this study, we used molecular docking and molecular dynamics simulations to explore the potential activity of the antiparasitic drug ivermectin (IVM) to prevent the binding of the SARS-CoV-2 SP to fibrinogen and reduce the occurrence of microclots. Our computational results indicate that IVM may bind with high affinity to multiple sites on the fibrinogen peptide, with binding more likely in the central, E region, and in the coiled-coil region, as opposed to the globular D region. Taken together, our in silico results suggest that IVM may interfere with SP–fibrinogen binding and, potentially, decrease the formation of fibrin clots resistant to degradation. Additional in vitro studies are warranted to validate whether IVM binding to fibrinogen is sufficiently stable to prevent interaction with the SP, and potentially reduce its thrombo-inflammatory effect in vivo.

## 1. Introduction

Fibrinogen, a large soluble glycoprotein, is the most abundant blood coagulation factor. It is converted to fibrin, the insoluble polymer scaffolding for blood clots, through a progressive series of reactions. Blood clots are involved in hemostasis, the protective response that stops bleeding. An alteration in their structural and mechanical properties can be associated with bleeding or, conversely, with pathological thrombosis that obstructs blood vessels, leading to often severe outcomes [1].

A recent study reported that the binding of the severe acute respiratory syndrome coronavirus 2 (SARS-CoV-2) spike protein (SP) glycoprotein to fibrinogen induces structurally abnormal, inflammatory blood clots that are common in asymptomatic, convalescent, and acute COVID-19 patients alike [2]. This binding is of clinical significance since the presence of SP has been demonstrated in human blood and tissue cells during acute infection [3] and for months afterwards in patients experiencing post-acute sequelae of COVID-19 (PASC) [4,5,6]. PASC, also known as long COVID, may affect 10 to 30% of SARS-CoV-2 infected individuals and is characterized by a myriad of long-term symptoms including but not limited to breathlessness, “brain fog”, inflammation, coagulopathies, unrelenting fatigue, neurological symptoms such as loss of smell and taste, tinnitus, and joint or muscle pain, which may become worse after physical exertion and last for at least 3 months after the acute infection [7,8].

Multiple research groups, including our own, have recently demonstrated that the macrocyclic lactone ivermectin (IVM) is able to bind competitively to the SP and limit its attachment to its host cell targets [9]. Based on these results, taken together with the reported binding between fibrinogen and the SP, we hypothesize that IVM could have similar binding activity with fibrinogen. The present study investigates binding sites and modes of IVM to fibrinogen through molecular docking and molecular dynamics simulations to predict its potential ability to limit SP–fibrinogen binding and potentially reduce microclotting in PASC patients. We have executed protein–protein docking simulations to identify which residues are involved in SP–fibrinogen binding and assess whether IVM could prevent the binding by interacting with the same residues on fibrinogen.

### 1.1. Structure and Function of Fibrinogen

**Figure 1:**
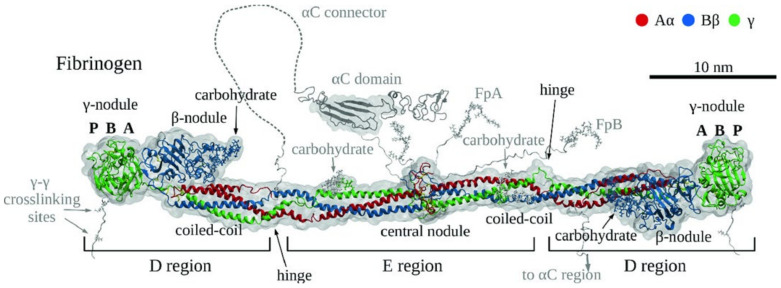
3D structure of fibrinogen. The Aα, Bβ, and γ chains are represented in red, blue, and green, respectively, while the computationally modeled missing regions are shown in grey. The location of carbohydrate ligands and hinge points is highlighted. P, B, and A correspond to the C-terminal A-domain, central B-domain and N-terminal P-domain in the γ-nodules. Reprinted with permission from Springer Nature (Litvinov et al., 2021 [1]). 2023, Springer Nature.

Human fibrinogen is a 340 kDa glycoprotein composed of two symmetrical halves, as shown in Figure 1. Each half consists of the Aα, Bβ, and γ chains. The N-termini of the α and β chains are called fibrinopeptides (Fp) A and B, respectively, and are cleaved by the enzyme thrombin to form the α and β chains in fibrin. The structure of about 66% of the human fibrinogen molecule has been resolved by X-ray crystallography (Protein Data Bank, or PDB, entry 3GHG) at a resolution of 2.90 Å [10]. Some unresolved motifs, including part of the αC domain and part of the β chain N-terminal domain [1,11], have been modeled in silico [11,12]. In the most recent modeling study, fibrinopeptides A and B and other regions of the molecule, such as residues 17–26, 231–412, 473–538, and 602–625 on the α chain, 15–20 and 55–57 on β, and 395–411 on the γ chain, were omitted because they are highly flexible and the study detected no structural information to guide their modeling [11].

#### Fibrinogen Domains and Interactions

Fibrinogen is a 45 nm long, rod-shaped molecule consisting of two lateral D regions and a central E region. Each D domain contains a globular part consisting of the β- and the γ-nodules and a coiled-coil portion connecting it with the E region’s central nodule. The β-nodule and the γ-nodule each consist of three domains: N-terminal A, central B, and C-terminal P domains.

Each domain contains binding sites mediating the various molecular interactions of fibrinogen. The E region contains the fibrinopeptides FpA and FpB that are enzymatically cleaved by thrombin in the first step of the blood clotting process. The release of FpA exposes a tripeptide motif at the N-terminus of the α chain (‘knob A’) that binds to the complementary ‘hole a’ located in the γ-nodule of another fibrin molecule’s D domain during polymerization. Similarly, the release of FpB exposes the ‘knob B’ in the E region that binds to ‘hole b’ in the β-nodule of the D domain, contributing to the lateral aggregation of protofibrils. Other structures that are thought to mediate the lateral assembly of protofibrils are the C-terminal region of the α (αC domains) and γ chains, the coiled-coils connecting the D and E domains, and the carbohydrate moieties associated with the β-nodules and the coiled portion of the γ chains.

Additional molecular interactions occur with other proteins, such as the plasma transglutaminase (factor XIIIa, activated from factor XIII in the presence of calcium ions Ca^2+^), which catalyzes covalent crosslinking of fibrin starting in the D domain (‘γ–γ crosslinking sites’ in Figure 1).

### 1.2. Fibrin Clot Formation

**Figure 2:**
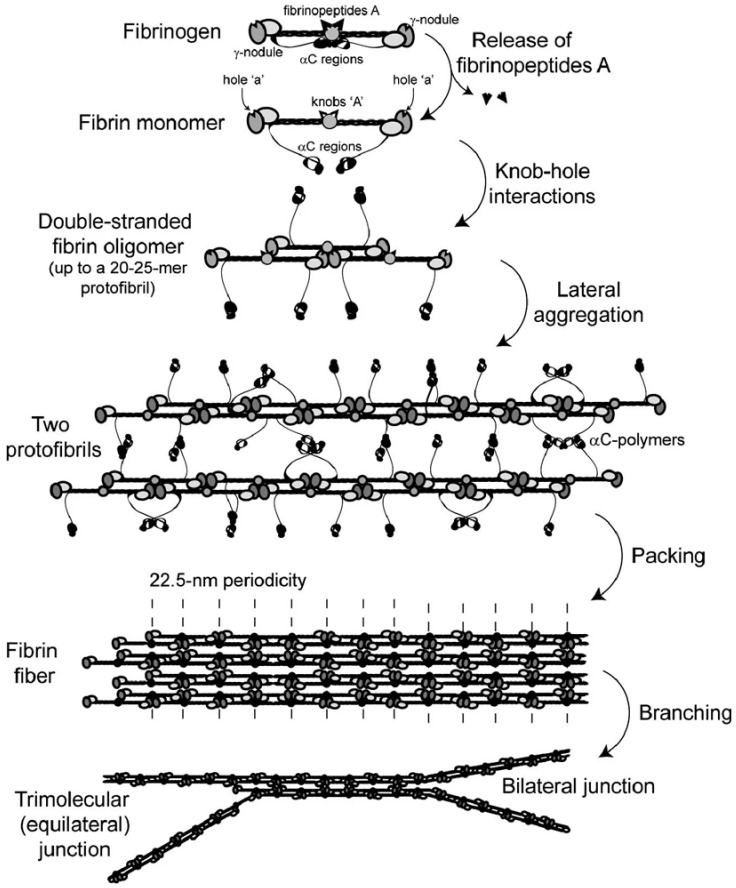
Schematic of the fibrin polymerization process. Reprinted with permission from Springer Nature (Litvinov et al., 2021 [1]). 2023, Springer Nature.

Figure 2 provides a schematic diagram of the fibrin polymerization process, which consists of a series of reactions:

1. Formation of monomeric fibrin: thrombin catalyzes the release of the FpA from the Aα chains, resulting in a fibrin monomer and exposing the ‘knobs A’. The release of FpB is a slower reaction, not essential for polymerization.
2. Formation of fibrin protofibrils: the fibrin monomers self-assemble so that the ‘knobs A’ in the E region of one monomer fit into two ‘holes a’ in the D domains of two other adjacent monomers. Longitudinal growth leads to the formation of double-stranded protofibrils.
3. Formation of fibrin fibers: protofibrils keep growing linearly and aggregate laterally at the same time to build a fiber, stabilized by ‘knob B–hole b’ bonds and other interactions.
4. Formation of a fibrin network: a three-dimensional network architecture is achieved through further longitudinal and lateral growth, as well as branching.

Covalent crosslinking by factor XIIIa occurs consistently during and after polymerization, a process which results in a porous, insoluble hydrogel-like network.

### 1.3. Clot Properties and Disease

An essential functional characteristic of clots is that in hemostasis, in order to stop the bleeding, the clot must be strong enough to resist the force of arterial blood flow. An altered clot with a looser, more porous and less rigid architecture has been associated with bleeding in coagulation disorders and hemophilia. In thrombosis, the mechanical properties of the thrombus determine whether the pressure of the blood flow will cause it to deform, dislodge, or rupture and embolize, and how it responds to treatment [1,13].

Various cardiovascular and other diseases involve alterations of the fibrin network architecture. Evidence of stiff clots with thin, closely packed fibers, yielding small pores and heightened resistance to fibrinolysis, has been found in relation to arterial and venous thrombosis, atherosclerosis, and chronic inflammatory diseases.

Notably, COVID-19 [14,15,16,17,18] and PASC (long COVID) [19] are characterized by the formation of microclots that are made by an anomalous amyloid form of fibrin, meaning they are non-porous deposits formed by densely matted fibers. These clots contain an ordered β-sheet architecture that gives them a characteristic affinity to fluorogenic amyloid stains (such as Congo red dye) and a resistance to proteolysis [20,21].

#### 1.3.1. Role of SP in Microclotting

**Figure 3:**
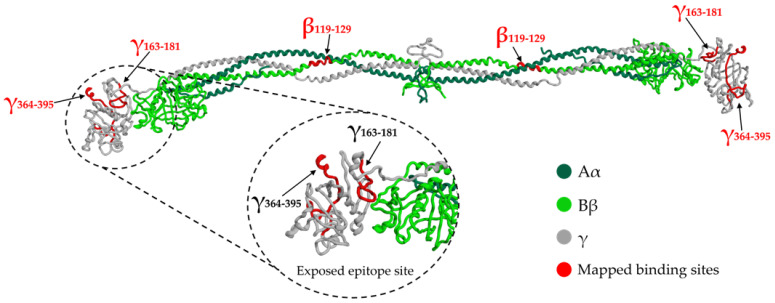
SP binding sites mapped on the fibrinogen structure (PDB entry 3GHG), as identified by Ryu et al., 2021 [2]. Illustration obtained in MOE v2022.02.

It has recently been observed that the cause of the formation of these anomalous blood clots can be traced back directly to the SARS-CoV-2 SP [2,22]. In particular, Ryu et al., 2021 proposed SP binding to fibrinogen and fibrin as a mechanism of action and identified three binding sites in the Bβ and γ fibrinogen chains [2], shown in Figure 3.

The sites mapped on the γ chain are part of the globular D domain: the γ_364–395_ site encompasses the γ_377–395_ binding site for complement receptor 3 (CR3), a member of the β2 integrin family that mediates innate immune response functions [23,24]; the function of the γ_163–181_ peptide is unknown. The binding site on the Bβ chain contains cleavage sites for the fibrinolytic protein plasmin. These findings can contribute to explaining the altered degradation and inflammatory properties of SP-associated fibrin clots.

Ryu et al., 2021 tested a monoclonal antibody generated for the γ_377–395_ epitope. They found that it suppressed inflammation without affecting normal hemostasis, suggesting that pharmacological targeting of the γ_377–395_ motif inhibits the pro-inflammation action of the SP [2].

#### 1.3.2. Potential Anti-Thrombo-Inflammatory Action of IVM

IVM is an orally bioavailable drug belonging to the avermectins, a group of macrocyclic lactones derived from the fermentation products of the soil bacterium *Streptomyces avermitilis* [25]. Approved for human use as an antiparasitic drug by the US Food and Drug Administration in 1987 [26], it has been used in 3.7 billion human doses since then [27] and has been proven safe at doses much higher than its standard dose of 200 μg/kg [28,29]. Its discovery and successful containment of two devastating global tropical diseases was recognized with the Nobel Prize for Medicine in 2015, with its safety record specifically noted in the announcement of that award [30]. A recent in silico study has predicted strong or moderate affinity binding of IVM and several related compounds to multiple sites on the SP, which can potentially interfere with its binding to the host cells [9]. In light of these findings, the present study explores the hypothesis that docking of IVM on fibrinogen in the binding sites of SP may prevent SP–fibrinogen binding and reduce microclotting.

### 1.4. Overview of Fibrinogen Binding Sites

Because of its multifunctional activity, interactions between fibrinogen and various small molecules have been reported. Table 1 provides an overview of the identified binding sites, compared with the results of the Site Finder application in the Molecular Operating Environment (MOE) 2022.02 [31].

Additionally, fibrinogen is involved in several protein–protein interactions (PPI). Table 2 provides a summary of the PPI sites compared with the results of the Site Finder application in the MOE 2022.02 software.

### 1.5. Structure of IVM

**Figure 4:**
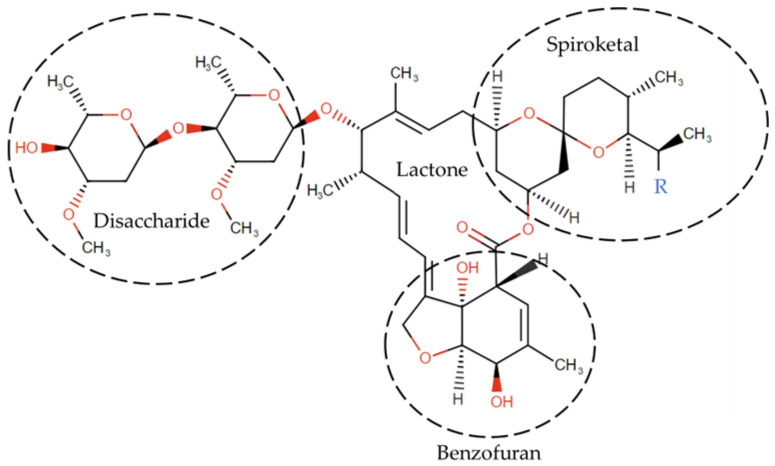
The structure of IVM is divided into the disaccharide, the lactone, the spiroketal, and the benzofuran regions. The substituent R in the spiroketal region is -CH_2_-CH_3_ in IVM_a and -CH_3_ in IVM_b.

IVM is a mixture of two avermectins, comprising roughly 90% 5-O-demethyl-22,23-dihydroavermectin A1a (22,23-dihydroavermectin B1a) and 10% 5-O-demethyl-25-de(1-methylpropyl)-22,23-dihydro-25-(1-methylethyl)avermectin A1a (22,23-dihydroavermectin B1b) [40], referred to as IVM_a and IVM_b, respectively, in the following. Its structure is depicted in Figure 4.

## 2. Results

### 2.1. Molecular Docking

**Figure 5:**
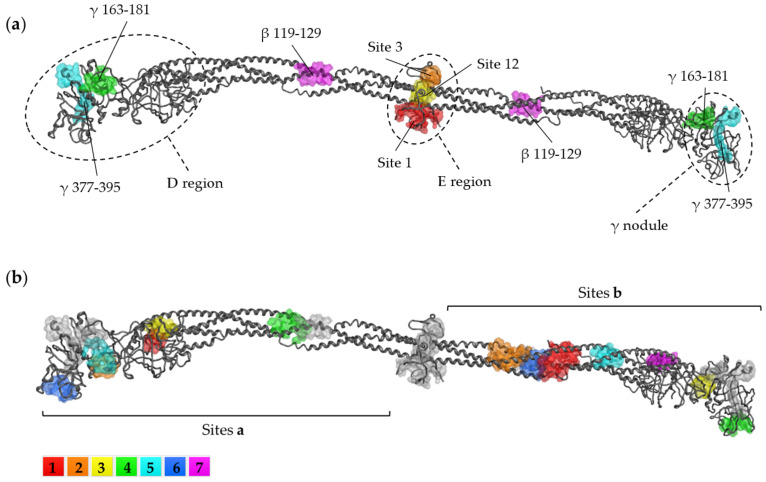
Location of the investigated binding sites on the fibrinogen structure. (**a**) E region and sites for SP binding; and (**b**) sites identified by MOE Site Finder on the two halves of the structure, numbered from 1 to 7 according to their ranked propensity for ligand binding (PLB) score as provided by the MOE 2022.02 Site Finder application.

IVM_a and IVM_b were docked to fibrinogen in the binding sites depicted in Figure 5.

Table 3, Table 4 and Table 5 list the docking scores in the E region, in the MOE Site Finder sites, and in the SP binding sites, respectively.

IVM_a has similar or higher docking scores than IVM_b in most cases. From the analysis of the docking results, it emerges that IVM seems to have a preference for the central E region of fibrinogen, site 1 in particular, that was also investigated with molecular dynamics (MD). Docking in site 1b (Table 4) also resulted in high scores; this pose was not simulated since it is located in the coiled-coil region, deemed too flexible to isolate. Analysis of the interactions did not reveal any evident pattern, though it was observed that all the investigated poses just participated in hydrogen bonds.

**Figure 6:**
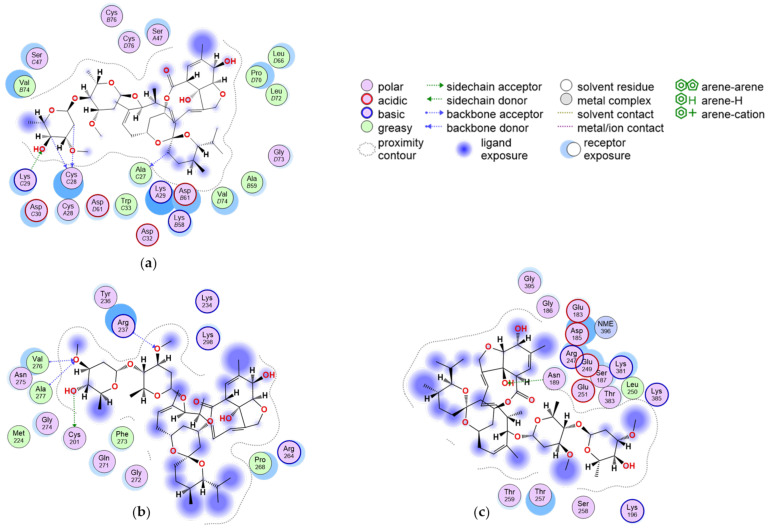
(**a**) Interactions of the docked pose of IVM_a in Site 1 of the central E region; (**b**) interactions of the docked pose of IVM_a in site gamma1; and (**c**) interactions of the docked pose of IVM_a in site gamma2.

The best-scoring poses that formed at least an interaction in Site 1 of the E region and in the predicted SP binding sites in the D region were used as input for the subsequent MD simulations. Their interactions with fibrinogen are summarized in Figure 6.

### 2.2. Molecular Dynamics

#### 2.2.1. E Region

**Figure 7:**
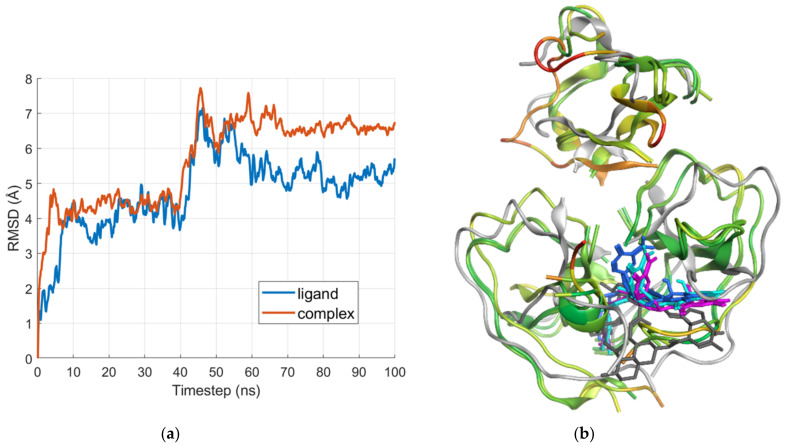
(**a**) RMSD plots of the E region in complex with IVM_a and the ligand alone; and (**b**) visual comparison of the docked pose (gray) with the averaged poses from the three largest clusters. The pose resulting from the most populated cluster is represented in magenta, the second-largest in cyan, and the third-largest in blue. The averaged structures of the clusters are color-coded according to the RMSD between them: green for low values, and red for high values.

Figure 7a shows the root-mean-square deviation (RMSD) plots of the E region in complex with IVM_a and of the ligand alone. The averaged poses of the three most populated clusters are compared with the docking result in Figure 7b.

As shown in Figure 7b, the ligand does not fully retain the space occupied by the docked pose, but buries itself further into the binding site. While both the spiroketal and disaccharide portions of IVM_a are involved in interactions in the docked pose, just the disaccharide region interacts in the clustered poses. The analysis of the interactions revealed that both the docked pose and all three clusters formed hydrogen bonds with the residue CYS3 in the N-terminal region of the α chain. The first cluster also made contact with ALA27 and LYS29, which are both involved in interactions with the docked pose as well.

Other residues involved in interactions in the MD snapshots are LYS58 and PRO60 in the β chain.

#### 2.2.2. D Region—Gamma1 Site

**Figure 8:**
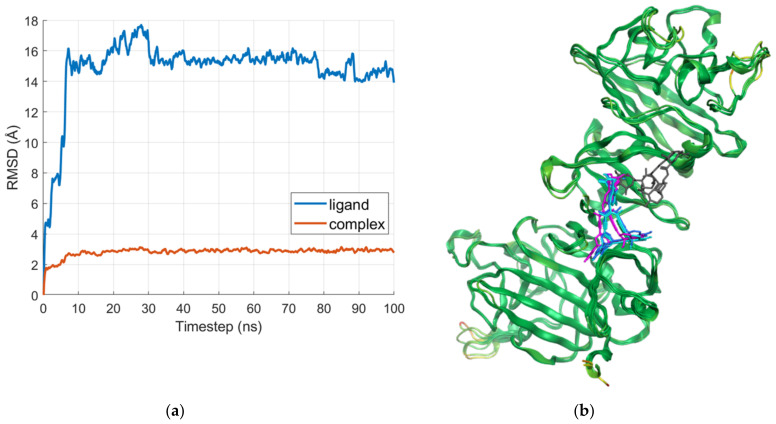
(**a**) RMSD plots of the D region in complex with IVM_a and the ligand alone; and (**b**) visual comparison of the docked pose (gray) with the averaged poses from the largest clusters. The pose resulting from the most populated cluster is represented in magenta, the second-largest in cyan, and the third-largest in blue. The averaged structures of the clusters are color-coded according to the RMSD between them: green for low values, and red for high values.

Figure 8a shows the RMSD plots of the D region in complex with IVM_a and of the ligand alone; the averaged poses of the most populated clusters are compared with the docking result in Figure 8b. The simulation shows a replacement of the ligand within the first 5–10 ns; therefore, the analysis of the interactions revealed no common residues between the docked pose and the clusters, although both interact with a cysteine residue.

#### 2.2.3. D Region—Gamma2 Site

**Figure 9:**
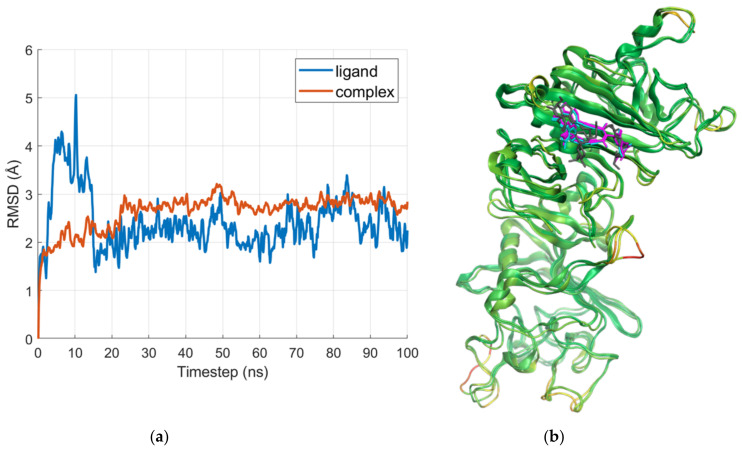
(**a**) RMSD plots of the D region in complex with IVM_a and the ligand alone; and (**b**) visual comparison of the docked pose (gray) with the averaged poses from the largest clusters. The pose resulting from the most populated cluster is represented in magenta, the second-largest in cyan, and the third-largest in blue. The averaged structures of the clusters are color-coded according to the RMSD between them: green for low values, and red for high values.

Figure 9a shows the RMSD plots of the D region in complex with IVM_a and of the ligand alone. The averaged poses of the most populated clusters are compared with the docking result in Figure 9b. In this case, the clustered poses overlap with the docked one, and formed more interactions than the docked pose, especially with aspartic acid and arginine residues.

### 2.3. Protein–Protein Docking

The interactions between fibrinogen and the free SP were predicted using the software PatchDock (http://bioinfo3d.cs.tau.ac.il/PatchDock/patchdock.html, accessed on 15 June 2023) [41]. The results were then refined and rescored using FireDock (http://bioinfo3d.cs.tau.ac.il/FireDock/firedock.html, accessed on 15 June 2023) [42]. We investigated both the closed state as represented by PDB entry 6VXX, and the open state, also known as the pre-fusion state with a single receptor-binding domain (RBD) up, which corresponds to PDB entry 6VSB.

The binding energy contributions of the top three refined and rescored outputs of FireDock are listed in Table 6 for the closed and open state of the SP.

**Figure A1:**
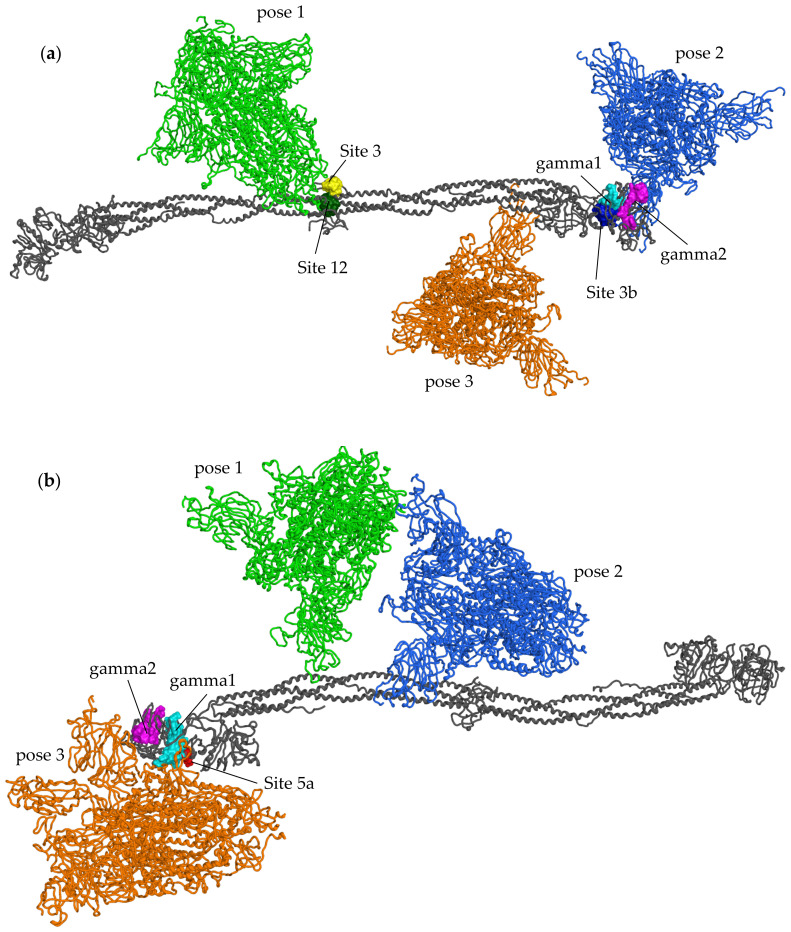
Depiction of the three top-scoring poses emerging from protein–protein docking of fibrinogen with both closed (**a**) and open (**b**) states of the SP. The fibrinogen molecule is colored in grey. The highest scoring pose of the SP in both states is illustrated in green, the second pose in blue, and the third in orange. The binding pockets where IVM inhibits fibrinogen, which happen to be situated at the interface of SP and fibrinogen, are emphasized as follows: Site 3 and Site 12, both located in the central E region, are highlighted in yellow and dark green, respectively. Meanwhile, the gamma1 site stands out in cyan, and the gamma2 site in purple; Site 3b, as identified by the Site Finder, is emphasized in dark blue, and Site 5a in red.

**Figure A2:**
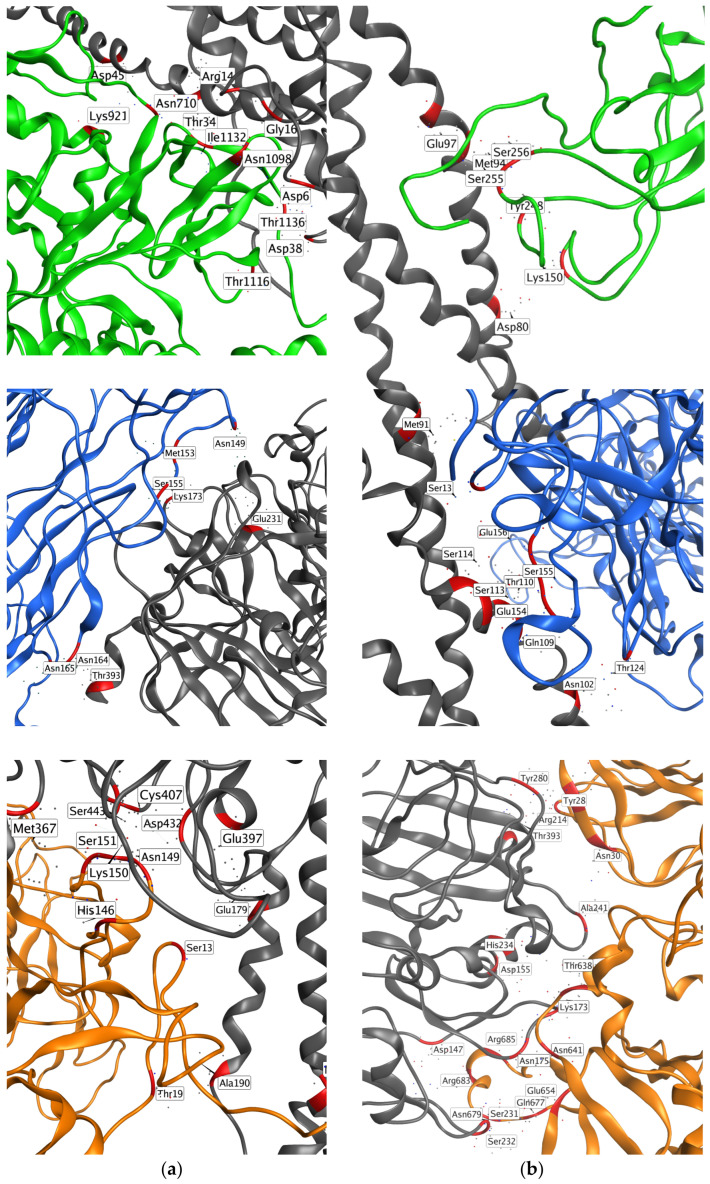
Contact residues at the interface of fibrinogen (depicted in grey) and the three top-scoring poses of the SP in its closed (**a**) and open (**b**) states. The highest scoring pose is illustrated in green, the second pose in blue, and the third place pose in orange. The residues participating in these interactions are prominently highlighted in red.

The most relevant interactions of the poses reported in Table 6 are listed below and illustrated in Figure A1 and Figure A2 in Appendix A.

Figure A1 emphasizes the IVM binding sites on fibrinogen, as previously demonstrated in Figure 5. It showcases that these sites are precisely located at the interface between SP and fibrinogen. This significantly supports our hypothesis: if we ascertain that the same residues implicated in the binding of SP and fibrinogen are also those inhibited by IVM on fibrinogen, we can infer that IVM could possibly interrupt the SP–fibrinogen interaction by interacting with these same residues on fibrinogen. As a result, this could potentially stave off the development of microclots.

Figure A2 effectively displays the contact residues at the interface of the SP and fibrinogen.

The following relevant interactions have been identified between fibrinogen and 6VXX (closed state SP):

1. The S2 subunit of the SP contacts Sites 3 and 12 in the central E region of fibrinogen. In particular, both the SP and the docked pose of IVM_a in Site 12, which had high affinity for fibrinogen in terms of docking score, are predicted to interact with two aspartic acid residues and an arginine residue in the γ chains, forming Site 12 (Asp27 in chain I and Asp6 and Arg14 in chain L);
2. The N-terminal domain (NTD) region of the S1 subunit of the SP contacts the γ chain of fibrinogen. Interacting residues belong to both the gamma1 (Leu172 and Lys173) and gamma2 (Phe389, Asn390, Thr393, Ile394) sites, and Site 3b (Lys173 and Glu231) from Site Finder;
3. Residues in the S1 subunit of the SP, neighboring the S2 cleavage site, contact chains α in the coiled-coil region and β in the globular D region.

Regarding 6VSB (pre-fusion state with a single RBD up), ithe NTD region of the two top poses contacts fibrinogen in the coiled-coil region. The third-best pose makes extensive contact with both the predicted SP binding sites in the γ chain of fibrinogen and with Site 5a from Site Finder. Specifically, it interfaces with residues Lys170, Leu172, Lys173, Ala174, Asn175, and Gln177 in the gamma1 site, along with residues Phe389, Thr393, and Ile394 in the gamma2 site. The docked pose of IVM_a in Site 5a, which yielded moderate docking scores (Table 4), also interacts with the Asn175 residue in the γ chain.

## 3. Discussion

Although the respiratory epithelium is typically the portal of infectious penetration of COVID-19, vascular abnormalities, including blood clots, constitute a major morbidity of this disease [43,44,45,46,47,48,49,50]. More generally, extensively damaged endothelium of pulmonary capillaries adjoining relatively intact alveoli has been observed in COVID-19 patients [51,52], which correspond to clinical symptoms of hypoxemia accompanied by normal breathing mechanics in such patients [47,49,50,51,53,54]. Another blood-based abnormality of COVID-19 related to, and perhaps a precursor to, blood clotting is the formation of clumps of red blood cells (RBCs), or rouleaux, as have been found prevalent in the blood of COVID-19 patients in three studies [55,56,57]. Even when loosely bound and reversible in their initial stages, rouleaux cause decreased efficiency of RBC oxygenation [57,58,59] and may be a key cause of decreased peripheral oxygen saturation, another major morbidity of COVID-19 [43].

The biochemical underpinning of rouleaux formation in the blood of COVID-19 patients is the binding of the SP glycoprotein of SARS-CoV-2 (as with the SP of most coronavirus strains) to host cell glycans, in particular, to those with terminal sialic acid (SA) moieties, which are densely distributed on RBCs and other blood cells [43]. In silico, IVM was shown to bind with high affinity to SARS-CoV-2 SP glycan sites [9], the implications of which were confirmed in a recent in vitro study [60]. Human RBCs mixed with SARS-CoV-2 SP caused hemagglutination (HA), while IVM added concurrently blocked HA, and IVM reversed HA when added after it had formed [60]. There are similarities between the formation of rouleaux and of fibrin-enmeshed blood clots, including the role of fibrinogen in each [61,62,63,64] (although hemagglutination occurs when RBCs and SP are mixed in the absence of fibrinogen [60]). It was therefore of interest whether IVM might be able to limit the development of blood clots in COVID-19 patients through inhibition of binding between blood cells, SARS-CoV-2 SP, and fibrinogen, analogous to its inhibition of SP-induced rouleaux formation. IVM binding to fibrinogen, if experimentally confirmed by in silico modeling and in vitro validation studies, may have important therapeutic implications for the large number of PACS patients suffering with prolonged and debilitating symptoms, since decreased formation of fibrin amyloid/SP microclots, which are able to block capillaries and limit the passage of red blood cells [2,17,18,19,20,21], may significantly increase oxygen diffusion and extraction in the peripheral microcirculation of PACS patients. Accordingly, in this study, we used molecular docking and molecular dynamics simulations to evaluate the capacity of IVM to bind to fibrinogen, the main clotting protein known to form “fibrinaloids” (i.e., fibrin amyloid microclots) resistant to fibrinolysis in the microcirculation of PACS patients [2,17,18,19,20,21].

Our in silico results comparing the docking scores of the two forms of IVM present in the clinical drug formulation demonstrated that IVM_a binds with a higher predicted affinity than IVM_b in most simulations. Of interest, we found IVM to bind preferentially to the central E region of fibrinogen, in particular to sites 1 and 12 (Table 3). IVM_a’s best pose was further investigated through molecular dynamics. It was observed that the ligand remained inside site 1 throughout the simulations, though did not retain the orientation of the docked pose, as shown in Figure 6.

The docking results in the Site Finder binding sites other than the E region (Table 4) indicated IVM’s predicted preference towards the coiled-coil regions of fibrinogen, though binding was generally not as strong as in the E region.

Docking in the SP binding sites (Table 5) yielded intermediate results in terms of binding scores; nevertheless, since these regions were predicted to interact with the SP, they were also simulated to evaluate the stability of IVM’s binding. In the case of the gamma1 site (centered on residues 163–181), the simulation shows a replacement of the ligand within the first 5 ns, before progressively stabilizing on the reached binding mode.

The analyzed simulations in the gamma2 site (centered on residues 377–395) show that the ligand tends to occupy the same space as in the docking simulation, although with more fluctuations and pose changes. Of interest, this binding area of IVM on fibrinogen overlaps with that of 5B8, a monoclonal antibody targeting the cryptic inflammatory fibrin epitope and able to inhibit thrombo-inflammation, as recently described by Ryu et al., 2021 [2].

The predictions derived from our protein–protein docking simulations exhibited some congruence with experimental findings by Ryu et al., 2021 [2]. Moreover, our simulations revealed a potential interaction site for the spike protein within the central E region of fibrinogen.

Our computational results suggest that IVM may bind with high affinity to multiple sites on the fibrinogen peptide, with binding more likely in the central, E region, in the coiled-coil region and in site gamma1, as opposed to site gamma2, in the globular D region. In light of the predicted interactions from protein–protein docking simulations, which show some overlap between the fibrinogen residues interacting with both the SP and IVM_a in the central E region and in the globular D region, these findings indicate, for the first time, the potential of IVM, an established, safe and widely used antiparasitic medication, to interfere with SARS-CoV-2 SP’s binding to fibrinogen. This interference may potentially decrease the formation of fibrin clots resistant to degradation, which may play a central role in the impaired oxygen extraction and systemic microvascular dysfunction reported in COVID-19 and PACS patients [65,66,67].

Additional in vitro studies will be necessary to assess whether IVM binding to fibrinogen is sufficiently stable to prevent interaction with the SP and potentially reduce its thrombo-inflammatory effect in vivo. Among these, binding assays to fibrinogen and albumin could be performed to evaluate whether albumin affects the bindings of IVM to fibrinogen, given that 93% of IVM binds to albumin in blood [68]. Other experimental testing can be sought to evaluate whether IVM lessens clot formation in the presence of SARS-CoV-2 SP, and if it does so at physiological concentrations.

## 4. Materials and Methods

### 4.1. Human Fibrinogen Structure

The PDB entry 3GHG representing a human fibrinogen structure is available in the Protein Data Bank [10]. It contains two copies of the fibrinogen structure; due to its lower number of missing residues, the second one was selected as a model. It is composed of chains G and J (corresponding to the Aα chains), H and K (Bβ), and I and L (γ), along with the co-complexed carbohydrates and calcium ions; it corresponds to the molecule as represented in Figure 1. A comparison between the numbers of missing residues of the two molecules in 3GHG is provided in Table 7.

We prepared the structure of human fibrinogen in MOE using the Structure Preparation panel. There were some challenges involving the unmodeled out-gaps in the C- and N-termini of all chains; however, no other regions needed addressing. Due to a limited number of viable templates, homology modeling of the out-gaps was not feasible. Meanwhile, the unconstrained nature of the termini introduced a wide range of possible conformations, making de novo modeling of the out-gaps a complex process. To navigate around these complexities, the out-gaps were removed from the termini and the terminus was subsequently capped for this preliminary study, laying the groundwork for future analysis. Note that the C-terminus out-gap on the gamma chain is in close proximity to the gamma 364–395 SP binding site identified by Ryu et al., 2021 [2]. The protein was protonated using the Protonate 3D application in MOE, and system settings include a pH of 7 and temperature of 300 K, while the default electrostatics model was used.

### 4.2. Molecular Docking

#### 4.2.1. Identification of Fibrinogen Binding Sites

The MOE Site Finder application was used to predict the most probable binding sites on the full structure of fibrinogen. This application employs a geometric method, implying that it does not depend on energy models for the identification of potential active sites in a receptor. The underlying concept is based on α-shapes, a family of piecewise linear curves in the Euclidean plane, which are intricately linked to the shape of a finite set of points [69]. The method identifies regions of tight atomic packing and proceeds to filter out unlikely sites such as protrusions, inaccessible regions, or too solvent-exposed ones. The potential sites are then ranked according to their propensity for ligand binding (PLB), based on their amino acid composition, which was implemented from [70]. The identified sites with a PLB score around or greater than 1 are listed in Table 8.

We utilized Sites 1, 3, and 12 to define the binding sites in the E region. To investigate sites beyond the E region in greater detail, we employed the Site Finder tool separately on the two halves of the fibrinogen structure. However, as the Site Finder application is primarily geared towards identifying small molecule binding sites, it may not fully encapsulate the sites for protein–protein interactions. As a result, the regions that interact with the SP were examined by positioning the docking box around the residues identified by Ryu et al., 2021 [2]. AMDock software v1.5.2 [71] was used to compute the optimal box placement with the “Center on Residues” option. Table 9 summarizes the investigated binding sites with the coordinates of the docking boxes, and Figure 5 maps their locations on the structure.

#### 4.2.2. Ligand Preparation

The ligand molecules were constructed from the SMILES strings obtained from DrugBank. The three-dimensional structures were prepared in MOE using the *Wash* procedure. Ligands were protonated as the most abundant protomers (dominant) at a pH of 7, hydrogens were explicitly added, and coordinates were calculated using the *Rebuild 3D* option.

#### 4.2.3. Molecular Docking Simulations and Analysis of Results

Molecular docking simulations were performed on each binding site listed in Table 9 by centering a cubic box of size 30 × 30 × 30 Å on the identified coordinates. The prepared structures of the receptor and ligands were converted to pdbqt format using the software OpenBabel v3.1.0 [72] before proceeding with docking using the software AutoDock Vina v.1.2.3 (latest stable) [73]. The exhaustiveness parameter was increased to 16 and the maximum number of output poses (num_modes) was increased to 20.

The docking results were analyzed in terms of predicted binding affinities (docking scores, expressed in kcal/mol) and ligand interactions with the receptor.

### 4.3. Molecular Dynamics Simulation

In an effort to mitigate the extensive computational demand typically associated with a comprehensive molecular dynamics simulation of a protein such as fibrinogen, which is large with a high aspect ratio, we isolated and simulated the D and E regions of interest, effectively ‘trimming’ the complex. This was accomplished by eliminating the coiled-coil region and subsequently capping the newly introduced termini. To model the D region, we utilized only one side of the symmetric fibrinogen structure; specifically, the H and I chains were selected as they presented smaller unmodeled out-gaps compared with the K and L chains. For Site 1 in the E region, we executed MD using IVM_a, due to its prominence as the most dominant form. In the D region, we carried out MD simulations for both the gamma1 and gamma2 sites, solely using IVM_a as the ligand (detailed binding site information can be found in Figure 5a and Table 9). The preferred pose for the ligand, used as the starting point in MD simulations, was determined by the most successful pose from molecular docking for each respective site. Our investigation encompassed a total of three site–ligand pairings. We prepared the protein structures and established the ligand poses using MOE, from which they were exported and set up for MD simulations through the MDKit Python package [74]. The MD protocol included a sequence of steps that included system solvation, two minimization stages, a heating stage, an equilibration stage, and, finally, a production stage. The stages of the protocol consisted of:

1. A minimization with positional restraints of 10 $\frac{\mathrm{kcal}}{\mathrm{mol}\cdot {Å}^{2}}$ applied to the heavy atoms of the solute;
2. A full-structure minimization step;
3. A heating phase up to 298 K in the NVT ensemble, using a Berendsen thermostat with a 2.0 picosecond coupling constant, τ;
4. An equilibration phase reaching 1.0 bar in the NPT ensemble, with constant pressure and temperature maintained with a Berendsen barostat (employing isotropic position scaling) and a Langevin thermostat, respectively. A pressure relaxation time of 2.0 ps was applied;
5. Finally, a 100 ns production run was performed.

The Amber20 software package [75] was used to run the MD simulations. Throughout all dynamic stages, a non-bonded cutoff distance of 10.0 Å and periodic boundary conditions were used. The structures were solvated in a truncated octahedron box with a minimum 10.0 Å distance from the protein to the box boundaries, employing a three-point water model (TIP3P). An ionic concentration of 0.15 M was achieved by adding appropriate amounts of sodium and chloride ions. The minimizations were carried out in two phases: initially, 5000 iterations of steepest descent, followed by 5000 iterations of conjugate gradient. The heating and equilibration phases each employed a time step of 2 fs and spanned 500 ps. The production runs adhered to the same settings as the equilibration stage, with the exception of returning the nonbonded skin to its default thickness of 2 Å. For each run, 1000 frames were saved to a trajectory file. The resulting trajectories were analyzed using the CPPTRAJ module of the Amber20 software package. The root-mean-square deviation (RMSD) was computed for each trajectory using the first frame as the reference. Subsequently, a clustering analysis was conducted to identify representative MD poses, facilitating comparison with the initial docked poses.

### 4.4. Protein–Protein Docking

The software PatchDock is a geometry-based algorithm aimed at finding good molecular shape complementarity [41]. It takes in two protein structures as input. In this scenario, fibrinogen serves as the “receptor” and remains stationary, while the SP is subjected to a sequence of transformations intended to align complementary surface patches on both proteins. The candidate transformations are evaluated and undergo an RMSD clustering analysis to eliminate any redundant results.

The outputs generated by PatchDock underwent additional refinement and re-scoring using FireDock [42]. The re-scored outputs were then ranked based on the global energy glob, or the binding energy of the docked pose, which encompasses both attractive and repulsive van der Waals energy (aVdW and rVdW) as well as atomic contact energy (ACE). We scrutinized the top three results from each simulation using the MOE Contacts application, focusing on factors such as hydrogen bonds, ionic bonds, arene interactions, covalent bonds, and distance interactions, associated with van der Waals distance interactions.

## 5. Conclusions

This in silico study follows up on recent experimental findings, which indicate that the SARS-CoV-2 SP may directly bind to fibrinogen and induce structurally abnormal blood clots with heightened proinflammatory activity. These blood clots are important features of both COVID-19 and PASC (long COVID), which are both characterized by hypercoagulability and the formation of microclots that can limit oxygen diffusion in the peripheral circulation, and can lead to symptoms including breathlessness, “brain fog”, inflammation, and fatigue, and lead to pathological thrombosis.

Several research groups, including our own, have recently demonstrated that ivermectin is able to bind competitively to the SP and limit its attachment to its host cell target, and we hypothesized that it could have similar binding activity with fibrinogen. Therefore, we undertook finding a molecular-level elucidation of the possible binding sites and modes of IVM on fibrinogen to predict its potential ability to limit SP–fibrinogen binding and potentially reduce microclotting. Our in silico analysis, utilizing both molecular docking and molecular dynamics simulations, indicates that IVM could bind with high affinity to the central E region of fibrinogen and with moderate-to-high affinity to the SP binding sites experimentally predicted by Ryu et al., 2021 [2]. Experimental testing of these computed binding affinities would help determine if IVM does indeed bind to fibrinogen, and, if so, whether it provides the indicated inhibition of SP-related microclotting.

## Figures and Tables

**Table 1:** Fibrinogen binding sites.

Ligand	Site	MOE Site Finder Results
Heparin	N-terminal regions (residues 15–57) of the two Bβ chains in the E region [32].	Not modeled
Flavonoids (myricetin, rutin, naringin, hesperidin, genistein, puerarin) and phenolic acids (ferulic and caffeic acid)	Hydrophobic cavity of E region near Trp 33 in the N-terminal region of the α chain [33]; residues 19–22 of the γ chain, residues 77–78 of the β chain [34].	Sites 1 and 12.
Gastrodin	Holes’ a’ and ‘b’ in the γ- and β-nodules of the D domain [35].	Identifiable with the position of the ligand chains Q and R for ‘a’ and S and T for the ‘b’ holes.
Benzothiazole and penicillin β-lactam antibiotics (cloxacillin and dicloxacillin)	Hydrophobic cavities of E region (bovine) [36,37].	Sites 1, 3, 12.

**Table 2:** Sites for protein–protein interactions of fibrinogen.

Interacting Protein	Site (s)	MOE Site Finder Results
Thrombin	Fibrinopeptides A and B in the N-termini of chains Aα and Bβ, in the central E region [1,38].	Not modeled.
Plasma transglutaminase	γ–γ crosslinking site, γ-nodule of D domain [1].	Not modeled.
Complement receptor 3	Residues 377–395 of the γ chain, γ-nodule of D domain [2].	Not modeled.
Subtilisin K2	Residue PHE117 on the Aα chain, LEU121 and TRP125 on the Bβ chain, and ASP53, PHE54, and THR57 on the γ chain [39].	Part of Sites 2, 11.
Plasmin	Residues 119–129 of the Bβ chain [2].	Part of Site 11.
SARS-CoV-2 SP	Residues 163–181 and 364–395 of the γ chain, in the γ-nodule of D domain; residues 119–129 of the Bβ chain [2].	γ: part of Sites 7 and 9. Bβ: part of Site 11.

**Table 3:** Best-scoring poses of IVM in the E region of fibrinogen.

Site	Score (kcal/mol)	
	IVM_a	IVM_b
Site 1	−10.971	−9.959
Site 3	−6.567	−6.426
Site 12	−10.142	−5.207

**Table 4:** Sorted scores for IVM in the sites identified by MOE Site Finder.

Site	Score (kcal/mol)	
	IVM_a	IVM_b
Site1b	−10.428	−9.506
Site4a	−9.202	−8.957
Site6b	−9.155	−8.223
Site3a	−8.820	−8.338
Site1a	−8.820	−6.385
Site5a	−8.800	−8.453
Site3b	−8.324	−7.875
Site7b	−8.288	−8.626
Site2a	−7.673	−8.056
Site4b	−7.058	−8.019
Site5b	−6.892	−6.802
Site6a	−6.844	−6.675
Site2b	−6.768	−7.039

**Table 5:** Sorted scores for IVM in the sites identified by Ryu et al., 2021 [2].

Site	Score (kcal/mol)	
	IVM_a	IVM_b
gamma1 (163–181)	−9.281	−8.915
gamma2a (377–395)	−7.136	−6.791
gamma2b	−7.448	−7.834
beta (119–129)	−9.921	−8.529

**Table 6:** Energies of the refined and rescored protein–protein docking results obtained with FireDock, reported in terms of global energy glob, attractive and repulsive van der Waals energy (aVdW and rVdW), and atomic contact energy (ACE).

Structure	Pose	glob	aVdW	rVdW	ACE
6VXX	1	−62.17	−65.02	38.94	4.11
	2	−49.83	−57.34	42.31	−4.01
	3	−46.34	−38.48	18.94	−3.99
6VSB	1	−54.06	−36.10	43.10	−9.79
	2	−55.15	−40.59	23.31	−3.11
	3	−35.48	−59.01	68.62	−1.01

**Table 7:** Missing residues in the 3GHG structures.

Chain	N-Terminus Out-Gap	C-Terminus Out-Gap
A (Aα)	26 residues	362 residues
B (Bβ)	57 residues	3 residues
C (γ)	13 residues	17 residues
D (Aα)	26 residues	362 residues
E (Bβ)	57 residues	3 residues
F (γ)	13 residues	16 residues
G (Aα)	26 residues	362 residues
H (Bβ)	57 residues	3 residues
I (γ)	1 residue	16 residues
J (Aα)	26 residues	350 residues
K (Bβ)	57 residues	3 residues
L (γ)	4 residues	16 residues

**Table 8:** Results of the MOE Site Finder with PLB around or over 1. Aα1 and 2 correspond to chains G and J, Bβ1 and 2 to chains H and K, and γ1 and 2 to chains I and L in the model. Boldened sites have correspondence, even partial, in the literature; green colored text indicates sites in the E region.

Site	PLB	Residues
**1**	**5.64**	**Aα1:(ACE26 ALA27 CYS28 LYS29 ASP32 TRP33 SER47 CYS49 ARG50)** **Bβ1:(LYS58 ALA59 PRO60 ASP61 CYS65 LEU66 HIS67 ASP69 PRO70 ASP71 GLY73 VAL74 LEU75 CYS76)** **Aα2:(ALA27 CYS28 LYS29 ASP30 SER31 TRP33 CYS45 PRO46 SER47 CYS49 ARG50 LEU54)** **Bβ2:(LYS58 ALA59 PRO60 ASP61 PRO70 ASP71 LEU72 GLY73 VAL74 CYS76)**
2	3.97	Aα2:(ILE93 ASP97 PHE98 ALA101 ASN102 ARG104 ASP105 ASN106 TYR108 ASN109 SER112 ARG116 HIS201 PRO203 LEU204 ILE205 MET207 LYS208 PRO211) Bβ2:(TRP125 ARG128 GLN129 VAL132 LYS133 ASN135 GLU136 VAL139 TYR142 SER143) γ2:(THR67 TYR68 ASN69 PRO70 ASP71 GLU72 SER73 LYS75 ASN77 MET78 ILE79 ASP80 THR83)
**3**	**2.50**	**γ1:(CYS8 CYS9 ILE10 TYR18)** **Bβ2:(CYS80 GLN83)** **γ2:(ACE4 ARG5 ASP6 CYS8 CYS9 SER17 TYR18 CYS19 PRO20)**
4	1.91	Aα1:(ILE156 SER160) Bβ1:(ARG255 ASP257 GLY258 SER259 VAL260 ASP261 PHE262 GLY263 GLU291 GLY399 ASN413 GLY414 ARG415 TYR416)
5	1.87	Bβ1:(PRO204 VAL206 LYS217 GLY218 GLY219 GLU220 THR221 GLU223 TYR225 TYR285) γ1:(VAL202 ASP203 LYS206 ILE209 GLN210 GLU213 GLY214 PHE215 GLY216 HIS217)
6	1.72	Aα2:(LEU73 TYR76 GLN77 ASN79 ASN80 LYS81 SER83 HIS84 THR87 THR88 MET91) Bβ2:(ASN103 VAL104 VAL107 SER108 THR110 SER111 SER114 PHE115 GLN116 TYR117 MET118) γ2:(LEU47 VAL50 GLU51 THR54 VAL57)
**7**	**1.63**	**Bβ2:(ILE203 PRO204 VAL205 VAL206 SER207 GLY208 LYS209 GLU213 LYS217 LEU226)** **γ2:(PRO171 LYS173 ALA174 ASN175 GLN176 GLN177 PHE178 LEU179 LYS212 GLU213 LEU218 LEU228 GLU231 LYS232 LEU235)**
8	1.38	A α1:(ASP153 ILE156 LYS157 SER160) Bβ1:(ASP261 GLY263 ARG264 TYR378 SER395 LYS396 GLY399 GLY400 GLY401 ARG415 LYS428 HIS429) γ1:(GLU132 GLN136)
**9**	**1.32**	**γ2:(PHE295 ASP297 ASP298 SER300 ASP301 PHE304 THR305 PHE322 CYS326 GLN329 ASP330 LYS338 CYS339 HIS340 TYR363 ASP364 ILE368 ARG375)**
10	1.20	Aα2:(ARG118 ILE119 LEU122 LYS123 LYS125 VAL126 LYS129 VAL130) Bβ2:(GLN150 ILE153 THR156 VAL157 ILE161) γ2:(ILE93 TYR96 GLU97 ILE100 HIS103 ASP104)
**11**	**1.08**	**A** **α** **2:(THR87 ILE90 MET91 LEU94 ARG95 PHE98)** **B** **β** **2:(TYR117 MET118 LEU121 LYS122 TRP125 GLN126 ARG128 GLN129)** **γ2:(LEU60 ILE61 ILE64 GLN65 THR67 TYR68)**
**12**	**0.99**	**Aα1:(CYS45 PRO46 SER47 GLY48)2:(CYS76 PRO77 THR78 GLY79)** **Bβ1:(CYS19 PRO20 THR21 THR22)** **Aα2:(CYS45 PRO46 SER47 GLY48)** **Bβ2:(CYS76 PRO77 THR78 GLY79 LEU82)** **γ2:(CYS19 PRO20 THR21 THR22)**
13	0.99	Aα2:(ASP153 LYS157) Bβ2:(ASP261 GLY263 ARG264 LYS265 TYR378 SER395 LYS396 GLY399 GLY400 ARG415 LYS428 HIS429) γ2:(LYS125 VAL128 ALA129 GLU132 ALA133 GLN136)

**Table 9:** Summary of the investigated binding sites.

Site	Site ID	Box Center Coordinates (Å)		
		x	y	z
E region	1	90.12	−53.03	−92.61
	3	97.22	−28.67	−88.12
	12	91.72	−38.97	−92.59
Chains G, H, I	1a	−62.20	−34.62	−45.53
	2a	−90.42	−49.52	−36.75
	3a	−58.83	−27.75	−44.12
	4a	10.65	−26.00	−83.01
	5a	−90.47	−42.66	−22.91
	6a	−114.10	−62.45	−34.73
Chains J, K, L	1b	173.90	−45.87	−107.26
	2b	142.69	−43.59	−96.29
	3b	272.20	−62.61	−166.68
	4b	292.84	−88.07	−160.55
	5b	203.59	−44.32	−114.73
	6b	158.42	−46.21	−103.52
	7b	241.40	−46.07	−144.96
SP-chain I	gamma1 (163–181)	−78.32	−29.61	−37.44
	gamma2a (377–395)	−99.64	−29.75	−41.74
	gamma2b ^1^	−107.40	−31.20	−44.80
	beta (119–129)	4.08	−28.05	−83.39

^1^ Centered on the pose showing the most interactions in site gamma2a.

## Data Availability

All data generated or analyzed during this study are available from the corresponding authors upon request.

## Abbreviations

The following abbreviations are used in this manuscript:

ACE	atomic contact energy
aVdW	attractive van der Waals energy
CR3	complement receptor 3
Fp	fibrinopeptide
fs	femtosecond
HA	hemagglutination
IVM	ivermectin
MD	Molecular dynamics
ns	nanosecond
NTD	N-terminal domain
PASC	post-acute sequelae of COVID-19 (long COVID)
PDB	Protein Data Bank
PLB	propensity for ligand binding
PPI	protein–protein interaction
ps	picosecond
RBC	red blood cell
RBD	receptor-binding domain
RMSD	root-mean-square deviation
rVdW	repulsive van der Waals energy
SA	sialic acid
SARS-CoV-2	severe acute respiratory syndrome coronavirus 2
SP	spike protein
